# Identifying key factors for the effectiveness of pancreatic cancer screening: A model‐based analysis

**DOI:** 10.1002/ijc.33540

**Published:** 2021-03-25

**Authors:** Brechtje D. M. Koopmann, Femme Harinck, Sonja Kroep, Ingrid C. A. W. Konings, Steffie K. Naber, Iris Lansdorp‐Vogelaar, Paul Fockens, Jeanin E. van Hooft, Djuna L. Cahen, Marjolein van Ballegooijen, Marco J. Bruno, Inge M. C. M. de Kok

**Affiliations:** ^1^ Department of Public Health Erasmus MC, University Medical Center Rotterdam Rotterdam The Netherlands; ^2^ Department of Gastroenterology & Hepatology Erasmus MC, University Medical Center Rotterdam Rotterdam The Netherlands; ^3^ Department of Gastroenterology & Hepatology Amsterdam University Medical Centers, University of Amsterdam Amsterdam The Netherlands

**Keywords:** microsimulation model, pancreatic cancer, screening

## Abstract

Pancreatic cancer (PC) survival is poor, as detection usually occurs late, when treatment options are limited. Screening of high‐risk individuals may enable early detection and a more favorable prognosis. Knowledge gaps prohibit establishing the effectiveness of screening. We developed a Microsimulation Screening Analysis model to analyze the impact of relevant uncertainties on the effect of PC screening in high‐risk individuals. The model simulates two base cases: one in which lesions always progress to PC and one in which indolent and faster progressive lesions coexist. For each base case, the effect of annual and 5‐yearly screening with endoscopic ultrasonography/magnetic resonance imaging was evaluated. The impact of variance in PC risk, screening test characteristics and surgery‐related mortality was evaluated using sensitivity analyses. Screening resulted in a reduction of PC mortality by at least 16% in all simulated scenarios. This reduction depended strongly on the natural disease course (annual screening: −57% for “Progressive‐only” vs −41% for “Indolent Included”). The number of screen and surveillance tests needed to prevent one cancer death was impacted most by PC risk. A 10% increase in test sensitivity reduced mortality by 1.9% at most. Test specificity is important for the number of surveillance tests. In conclusion, screening reduces PC mortality in all modeled scenarios. The natural disease course and PC risk strongly determines the effectiveness of screening. Test sensitivity seems of lesser influence than specificity. Future research should gain more insight in PC pathobiology to establish the true value of PC screening in high‐risk individuals.

## Abstract 2

What's new?

About 10 percent of pancreatic cancers occur in individuals with inherited risk factors. While screening such high‐risk individuals can facilitate the detection of precursor lesions and early‐stage cancer, the extent to which benefits outweigh harms, including overdiagnosis, remains unknown. Here, using a microsimulation model, the authors explored uncertainties concerning the early detection of pancreatic cancer and analyzed the impact of these uncertainties on the effect of screening. In all simulated scenarios, screening was associated with reduced pancreatic cancer mortality. The effectiveness of screening was most strongly impacted by characteristics of natural disease course and level of pancreatic cancer risk

**Abbreviations**
EUS: endoscopic ultrasonography
HGD: high‐grade dysplasia
IGD: intermediate‐grade dysplasia
LGD: low‐grade dysplasia
LYG: life‐years gained
MISCAN: Microsimulation Screening Analysis
MRI: magnetic resonance imaging
NNS: number needed to screen
NNSurv: number needed to surveil
NNT: number needed to treat
PanIN: pancreatic intraductal neoplasia
PC: pancreatic cancer

## 1 INTRODUCTION

Pancreatic cancer (PC) has one of the poorest survival rates of all human cancers and is ranked among the top five of cancer‐related deaths.^1^ Although the general incidence is low (lifetime risk of 1.3%), it is substantially increased in certain high‐risk groups, with relative risks up to 132 in Peutz Jeghers Syndrome, for instance.^1^ Approximately 10% of PC cases are found in individuals with an inherited increased risk for this fatal disease. This high‐risk population includes carriers of PC‐prone gene mutations (eg, *BRCA2*, *CDKN2A* and *LKB1*) and first‐degree relatives of familial PC patients. Familial PC patients have at least (a) one first‐degree relative with PC, (b) one second‐degree relative <50 years at time of PC diagnosis or (c) two relatives with PC without a known gene mutation.^1^

Several studies have shown that screening individuals at high risk for PC can detect precursor lesions and early stage cancer.^1^ However, we lack solid evidence that the benefits of screening (reduced PC‐related morbidity and mortality) outweigh its harms, such as patient burden, overdiagnosis and overtreatment. Determination of the effectiveness of screening requires large‐scale and prolonged follow‐up.

Computer simulation models have proven their value in estimating the long‐term impact of screening using short‐term indicators such as PC incidence and detection rates. The Microsimulation Screening Analysis (MISCAN) model, for example, has been used for the evaluation and optimization of screening programs for cancer of the cervix, breast, colon, prostate, lung and esophagus.^2^, ^3^, ^4^, ^5^, ^6^, ^7^ This model has also been applied to test hypotheses on disease development^3^ and test characteristics.^4^

In order to create an accurate simulation model, detailed information on the disease is necessary. Current knowledge gaps in PC screening include the natural disease course, test characteristics (sensitivity, specificity) for different disease stages, and the PC risk level. Decision‐analytic modeling can be used to explore such uncertainties and quantify the impact of individual factors on the effect of screening.^2^, ^4^ Identification of the factors with the strongest impact on screening may guide future research.

In our study, we aim to identify key parameters that define the effect of PC screening and to analyze their impact on the effect of PC screening in a high‐risk population using the microsimulation model MISCAN. Consequently, we will identify areas to which further research should be directed.

## 2 METHODS

### 2.1 MISCAN‐pancreas model

The MISCAN model generates a large simulated study population with individual life histories. Each person can develop a preinvasive lesion that can evolve into PC, from which that person may die at a certain point in time. This simulation results in an age‐ and time‐specific output of disease incidence and mortality. The simulated population then undergoes PC screening, which will change life histories in case screening detects preinvasive lesions or early‐stage cancer that can be surgically resected. These changes constitute the effects of PC screening and are represented by the number of prevented cancer cases and a potential shift in stage distribution.

The stochastic model underlying the simulation is composed by input parameters. These parameters relate to the demographic characteristics (eg, the life table), epidemiology and natural disease course (eg, duration of the different disease stages) and screening characteristics (eg, the sensitivity of the screening test[s]). Since evidence‐based data are scarce in the setting of PC screening of high‐risk individuals, many assumptions are based on consensus recommendations, as stated by the international Cancer of the Pancreas Screening‐consortium.^1^

### 2.2 Base case analyses

#### 2.2.1 PC risk and natural disease course

We assume a lifetime risk of 7.5% for developing clinical PC, based on the current knowledge of PC risk in high‐risk individuals.^1^ In the model, the disease is subdivided into preinvasive and invasive stages. Preinvasive lesions (ie, pancreatic intraductal neoplasia (PanIN) and intraductal papillary mucinous neoplasm) are subdivided into three consecutive stages, based on grade of dysplasia; low‐, intermediate‐ and high‐grade (respectively LGD, IGD and HGD). Invasive lesions are graded according to TNM stages: I, II and III/IV.^8^ Stages III and IV are simulated together because treatment and survival are comparable. While preinvasive lesions can only be detected by screening or surveillance, invasive stages can also be detected clinically (ie, because of symptoms).

The natural disease course of PC, in particular the duration of the different preinvasive stages, is unknown. Therefore, we model two base cases with contrasting disease pathways, to look at the effect of different progression patterns on screening outcomes. In the first base case, we simulate a path where all lesions progress from preclinical disease to clinical cancer, in an average of 14.3 years, based on the scarce evidence available.^9^ We assume durations are exponentially distributed (Weibull distribution, Shape 1) and that the duration of each disease stage is correlated with the next: in other words, when a lesion is fast growing in one stage, it does so in the next. Durations varied based on random selection in the distribution. There are no indolent lesions in this base case.

Since the average dwelling time of 14.3 years is debated, we model a second base case, in which both faster progressive and indolent (slow developing) lesions are simulated. In case of slow developing disease, lethal PC will never occur in the course of a lifetime. Preinvasive stages will at most progress to preclinical cancer Stage I. This is assumed to take over 30 years. In this base case, the preinvasive lesions that do progress to preclinical cancer, progress much faster, in an average time of 5 years, compared to the other base case. More detailed information on the assumed natural disease courses is provided in Table 1 and in the Appendix.

**TABLE 1:** Model assumptions on the natural disease course of PC and its precursors

	Value	
Lifetime PC risk	7.5%^1^	
Treatment mortality	3%^10^, ^11^	
	*Progressive‐only pathway*	*Indolent Included pathway*
Stage	Mean duration of progressive stages (years)	
Preinvasive stage LGD	3.33	1.11
Preinvasive stage IGD	3.33	1.11
Preinvasive stage HGD	3.33	1.11
Preclinical cancer Stage I	2	0.66
Preclinical cancer Stage II	2	0.66
Preclinical cancer Stage III/IV	1	0.33
Estimated mean total preclinical	14.3	4.8
Stage	Mean duration of indolent stages (years)	
Preinvasive stage LGD	n.a.	7.08^a^
Preinvasive stage IGD	n.a.	11.78^a^
Preinvasive stage HGD	n.a.	24.15^a^
Preclinical cancer Stage I	n.a.	Until death from other causes
Stage	Probability of being clinically diagnosed, before moving to the next stage^12^ ^b^	
Cancer Stage I	5.1%	
Cancer Stage II	13.1%	
Cancer Stage III/IV	100%	
Stage	5‐year relative survival^12^ ^b^ ^,^ ^c^	
Clinical cancer Stage I	29.1%	
Clinical cancer Stage II	11.4%	
Clinical cancer Stage III/IV	0%	
Stage	Screen test sensitivity^d^	
Preinvasive stage LGD	60%	
Preinvasive stage IGD	60%	
Preinvasive stage HGD	75%	
Preclinical cancer Stage I	90%	
Preclinical cancer Stage II	93%	
Preclinical cancer Stage III/IV	99%	
	Screen test specificity^d^	
Any pancreatic lesion	90%	

*Notes*: If someone has survived the first 5 years after diagnosis, we assumed lifelong PC survival. Detection (and associated management of preinvasive lesions) was assumed to lead to a 100% cure rate. For resection, we assumed a 3% mortality risk. Stage‐specific survival of screen‐detected invasive cancer was based on observed survival of clinically detected cancer in the Netherlands.

^a^ Optimized parameter.

^b^ Weighted averages are used to combine cancer Stage Ia and Ib into Stage I, and IIa and IIb into Stage II.

^c^ A linear distribution of PC mortality was assumed over 5 years.

^d^ Combined MRI/EUS.

**FIGURE 1:**
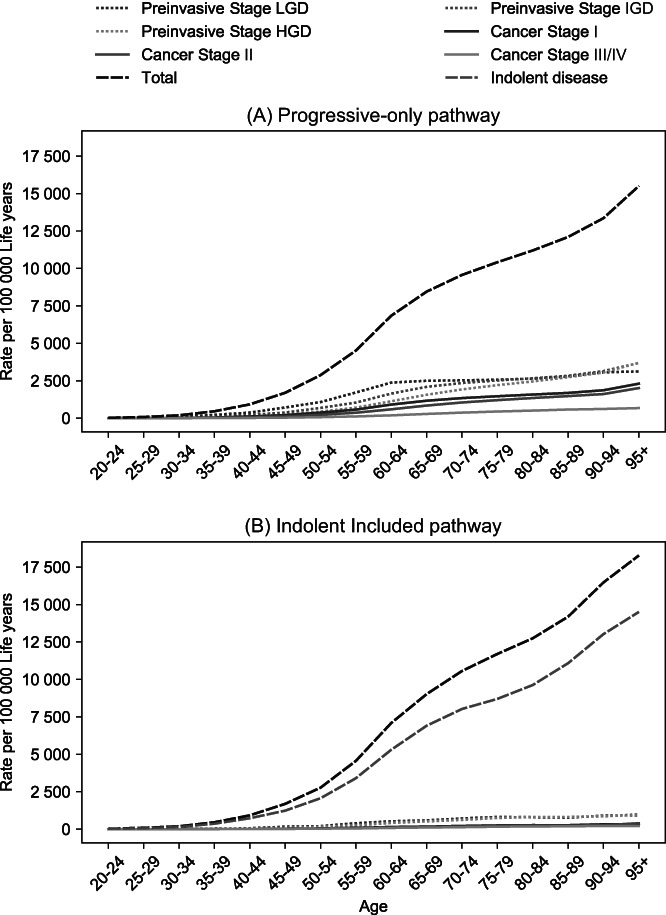
Estimated age‐specific prevalence of different disease stages in high‐risk individuals when modeling only progressive lesions (panel A), or modeling both indolent and faster progressive lesions (panel B), in the absence of screening

For both base cases, we use PC lifetime risk as a calibration target. We have created a high‐risk population by multiplying the PC lifetime risk of the general population by 10. Figure 1 shows the prevalence of each disease stage by age, for both the “Progressive‐only” and “Indolent Included” pathways.

### 2.3 Assumptions for screening, surveillance and treatment

**FIGURE 2:**
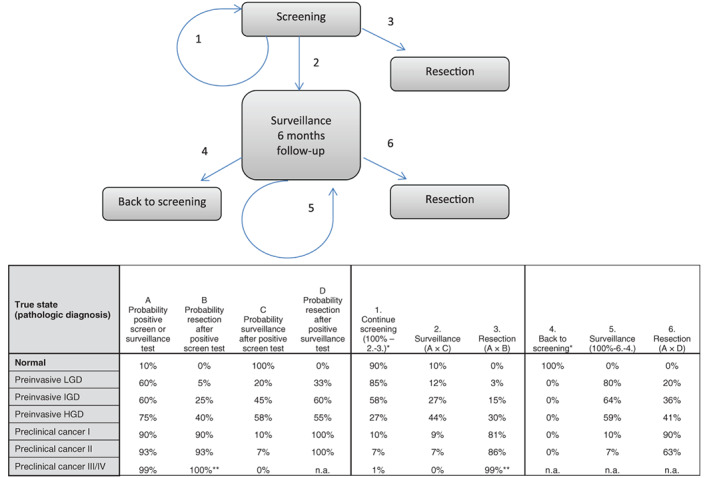
Screening strategy evaluated in the MISCAN‐pancreas model. Transition 1 (“continue screening”) is the effect of a (false‐) negative test result. Values in table are for the base case situation. *1‐ or 5‐year interval. **In case of cancer Stage III/IV, patients only receive palliative care (no resection) [Color figure can be viewed at wileyonlinelibrary.com]

For both base cases, we simulated a hypothetical cohort of 1 000 000 high‐risk individuals from birth until death with and without screening. When screening is implemented, these individuals are offered yearly or 5‐yearly screening from the ages of 50 to 75. Individuals are assumed to adhere to all screening rounds. The screen test characteristics are equivalent to a combination of both an endoscopic ultrasonography (EUS) and a magnetic resonance imaging (MRI), as they provide the most accurate pancreatic imaging.^1^ The screen test is considered positive when a preinvasive or invasive lesion is detected, rightfully or wrongfully. Surveillance tests are defined as additional tests, performed every 6 months after a positive screen test until the lesion was resected (Figure 2). Test characteristics of the surveillance test are assumed identical to those of the screen test.

As explained in the abovementioned paragraph on natural disease course, disease development occurs in different stages. We assume test characteristics differ between these stages, based on preliminary results of a PC screening study cohort (Figure 2). We assume that 90% of normal pancreata (no lesions) are correctly identified on imaging and 10% will be diagnosed as false positive. These individuals will be referred to more intensive follow‐up (surveillance test) or surgical resection. Figure 2 shows screen (and surveillance) test sensitivities for each disease stage. For individuals with a preinvasive lesion harboring LGD, IGD or HGD, tests are assumed to be positive in 60%, 60% and 75% of cases, respectively, and detection will result in surveillance or surgery. In case of preclinical cancer Stage I, II or III/IV, sensitivity is assumed to be 90%, 93% and 99%, respectively. Detection of preclinical cancer Stages I and II results in surgery. Individuals who are diagnosed with preclinical cancer Stage III/IV will receive palliative care.

Not all lesions detected by screening will develop into clinical cancer when left untreated. When detected, these clinically irrelevant findings may lead to overtreatment. In case of a positive screen test, individuals either undergo surgery (Figure 2, transitions 2 and 3) or surveillance. The proportion of patients that undergoes resection is determined by the test sensitivity and the probability that a positive‐tested patient undergoes resection, right or wrongfully so. This probability is based on different factors in the clinical process: risk of malignancy for the different precursor lesions, patient symptoms, changes over time, additional tests and multidisciplinary meetings (eg, test interpretation). For example, when 90% of individuals with preclinical cancer Stage I will have a positive screening test and 90% of these patients will undergo resection, 81% of all individuals with preclinical cancer Stage I will undergo resection. Some individuals return straight back to regular screening, based on the interpretation of the positive test result. Detection and associated management of preinvasive lesions was assumed to lead to a 100% cure rate (ie, no cancer development). However, such patients remain at risk for development of new pancreatic lesions. For resection, we assume a baseline 3% mortality risk.^10^, ^11^

### 2.4 Outcome parameters and sensitivity analyses

For each of the six scenarios (two base cases with and without annual or 5‐yearly screening), the model counts all relevant events, that is, the number of screening and surveillance tests, resections, cancer diagnoses and cancer deaths. From this, we can calculate cancer incidence and mortality rates, life‐years gained (LYG) and interval‐cancer rates (in the first 5 years and in the total period after screening). An interval cancer is defined as a clinically detected cancer after a negative screen test. For each scenario with screening, we calculate the number needed to screen (NNS), number needed to surveil (NNSurv) and number needed to treat to prevent one cancer death (NNT) as measures of effectiveness. We evaluated these measures of effectiveness in terms of preventing cancer death instead of detecting one cancer because of the deadly nature of PC.

**FIGURE 3:**
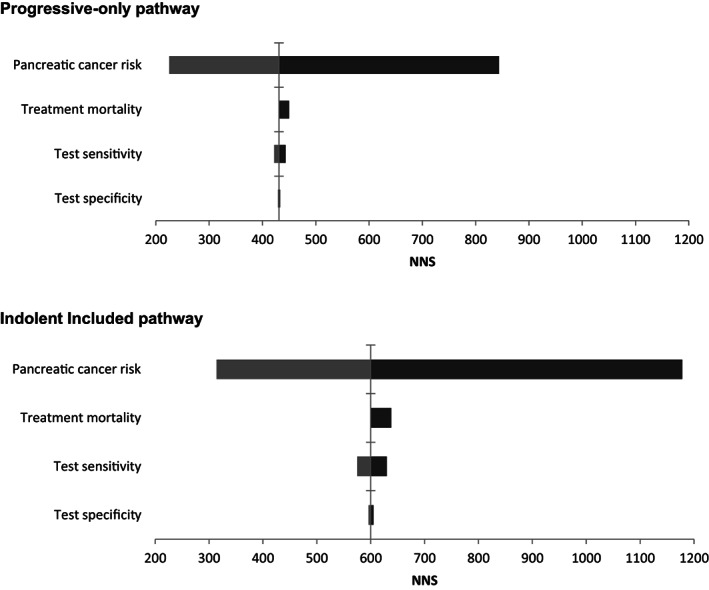
The effect on NNS of the sensitivity analyses for screening from ages 50 to 75, with a 1 year interval for both the Progressive‐only and the Indolent Included pathway. NNS is the number of screening tests needed to prevent one cancer death. Vertical line: base case result. Light grey bars: PC risk doubled, test sensitivity for each disease stage increased with 10% (110% of base case value), test specificity decreased to 85%. Dark grey bars: PC risk halved, mortality rate increased from 3% to 5%, test sensitivity decreased with 10% (to 90% of base case value), test specificity increased to 95%

Besides assuming different disease pathways and screen intervals, we estimate the impact of changes in other variables as PC risk, test performance and treatment‐related mortality on the effect of screening by sensitivity analyses (Figure 3 and Appendix Tables 2 and 3).

The lifetime risk for developing PC of 7.5% in the base cases is halved and doubled in the sensitivity analyses. Test specificity is decreased from 90% to 85% and increased to 95% and 100%. The assumed test sensitivity is varied by 5% (ie, 95% and 105% of its base case value) and by 10% (ie, 90% and 110% of base case value) for each disease stage, up to a maximum of 100%. Finally, we increase the treatment mortality risk from 3% to 5%.

## 3 RESULTS

### 3.1 Base case analyses; Progressive‐only pathway

Without screening, 921 PC cases and 751 PC deaths per 10 000 individuals were found using the model including only progressive disease. The mortality rate was 108 deaths per 100 000 life years (LYs).

Annual screening led to a 49% decrease in number of cancer‐cases, from 921 to 466 cases, and prevented 432 cancer‐deaths per 10 000 persons simulated. This corresponds with a 57% reduction in mortality rate, from 108 to 46 deaths per 100 000 LYs (Table 2).

**TABLE 2:** Base case results for screening at ages 50 to 75, with a 1‐ and 5‐year interval, per 10 000 simulated persons (ie, 9250 and 9251 screened individuals) for the Progressive‐only and the Indolent Included pathway

	BASE CASE Progressive‐only pathway					Base case Indolent Included pathway				
	No screening	1‐year interval		5‐year interval		No screening	1‐year interval		5‐year interval	
Disease burden (% reduction compared to no screening)										
Cancer cases	921	466 (−49.4%)		723 (−21.5%)		918	659 (−28.2%)		887 (−3.4%)	
Cancer deaths	751	319 (−57.5%)		495 (−34.1%)		753	444 (−41.0%)		624 (−17.1%)	
Incidence (per 100 000 LYs)	132	67 (−49.2%)		104 (−21.2%)		132	94 (−28.8%)		127 (−3.8%)	
Mortality (per 100 000 LYs)	108	46 (−57.4%)		71 (−34.3%)		108	64 (−40.6%)		89 (−17.6%)	
Screen/surveillance tests										
Number of screening tests	0	186 504		42 415		0	185 594		42 175	
Number of surveillance tests	0	20 182		4945		0	20 025		4941	
Resections										
Total number of resections (100%)	0	1233		846		0	1278		778	
Number of preinvasive stage LGD resections	0	292	(23.7%)	82	(9.7%)	0	286	(22.4%)	88	(11.3%)
Number of preinvasive stage IGD resections	0	414	(33.6%)	201	(23.8%)	0	391	(30.6%)	205	(26.3%)
Number of preinvasive stage HGD resections	0	233	(18.9%)	207	(24.5%)	0	254	(19.9%)	204	(26.2%)
Number of SD cancer Stage I resections	0	252	(20.4%)	256	(30.3%)	0	276	(21.6%)	203	(26.1%)
Number of SD cancer Stage II resections	0	40	(3.2%)	97	(11.5%)	0	66	(5.2%)	74	(9.5%)
Number of SD cancer Stage III/IV	0	2	(0.2%)	3	(0.4%)	0	5	(0.4%)	4	(0.5%)
Effectiveness										
LYs gained	0	4015		2291		0	3052		1233	
Interval cancers,^a^ total (per 100 000 LYs)	0	40		136		0	106		257	
NNS	n.a.	431		166		n.a.	600		326	
NNSurv	n.a.	46.7		19.3		n.a.	64.8		38.2	
NNT	n.a.	2.9		3.3		n.a.	4.1		6.0	

Abbreviations: LYs, life years; NNS, number needed to screen to prevent one cancer death; NNSurv, number needed to surveil; NNT, number needed to treat to prevent one cancer death; SD, screen detected.

^a^ The interval cancer rate is presented as the number of cancer cases (per 100 000 LYs) in the first 5 years after a negative screening test and in the total period after a negative screening test (including after age 75) (screen detected [SD] cancer cases after a negative screening test are not included).

Five‐yearly screening reduced mortality by 34%, compared to no screening. The NNS with yearly and 5‐yearly screening were 431 and 166, respectively. Annual screening led to more resections of LGD and IGD lesions than 5‐yearly screening (706 vs 283).

Five‐yearly screening resulted in less resected lesions harboring HGD, as compared to annual screening (207 vs 233). The number of resected cancer Stages I, II and III were 256, 97 and 3 with 5‐yearly screening and 252, 40 and 2 with annual screening. The NNT was 2.7 for yearly screening and 3.3 for 5‐yearly screening.

### 3.2 Base case analyses; Indolent Included pathway

Without screening, the model including both indolent and faster progressive disease, simulated 918 PC cases and 753 PC deaths per 10 000 individuals. The mortality rate was 108 deaths per 100.000 LYs.

Annual screening led to a 28% decrease in number of cancer‐cases, from 918 to 659 cases, and prevented 309 cancer‐deaths per 10 000 persons simulated. This corresponds with a 41% reduction in mortality rate, from 108 to 64 deaths per 100 000 LYs.

Five‐yearly screening reduced mortality by 18%, compared to no screening. The NNS with yearly and 5‐yearly screening were 600 and 326, respectively. Also, in this base case, the level of resected less invasive lesions (LGD and IGD) was higher when screening takes place annually, but to a lesser extent than in the Progressive‐only model. The number of resected HGD lesions was also lower in case of 5‐yearly screening compared to annual screening (204 vs 254). The number of resected cancer Stages I, II and III were 203, 74 and 4 with 5‐yearly screening and 276, 66 and 5 with annual screening. The NNT was higher in case of 5‐yearly screening, as compared to annual screening (6.0 vs 4.1).

### 3.3 Sensitivity analyses

The effect of different sensitivity analyses on NNS for both pathways in case of annual screening are visualized in Figure 3. An overview of all sensitivity analyses on different outcome measures is provided in Table 3 (annual screening) and in Appendix Tables 2 and 3 (5‐yearly screening).

**TABLE 3:** Results of the sensitivity analyses of the two base cases for annual screening

		Incidence (per 100 000 LYs)	Incidence reduction (%)	Mortality (per 100 000 LYs)	Mortality reduction (%)	NNS	NNT	NNSurv
Population without screening		132	n.a.	108	n.a.	n.a.	n.a.	n.a.
Progressive‐only pathway								
Base case		67	49.2%	46	57.4%	431	2.9	47
Risk	Halved	34	50%^a^	23	58.2%^b^	844	2.8	87
	Doubled	130	48.2%^a^	89	57.0%^b^	225	3.0	26
Sensitivity	+5%	66	50.0%	45	58.3%	427	2.8	46
	+10%	64	51.5%	44	59.3%	422	2.8	46
	−5%	68	48.5%	46	57.4%	437	2.9	47
	−10%	69	47.7%	47	56.5%	443	2.9	48
Specificity	85%	66	50%	45	58.3%	429	2.8	67
	95%	67	49.2%	46	57.4%	433	2.8	26
	100%	68	48.5%	46	57.4%	435	2.9	5
Treatment mortality	5%	67	49.2%	48	55.6%	450	3.0	49
Indolent Included pathway								
Base case		94	28.8%	64	40.7%	600	4.1	65
Risk	Halved	48	29.4%^a^	33	40.0%^b^	1178	4.1	122
	Doubled	182	27.5%^a^	123	40.6%^b^	314	4.3	37
Sensitivity	+5%	93	29.5%	63	41.7%	587	4.1	64
	+10%	92	30.3%	62	42.6%	575	4.1	62
	−5%	96	27.3%	65	39.8%	615	4.2	66
	−10%	97	26.5%	66	38.9%	630	4.2	68
Specificity	85%	94	28.8%	63	41.7%	596	4.1	93
	95%	95	28.0%	64	40.7%	605	4.1	36
	100%	96	27.3%	64	40.7%	613	4.2	7
Treatment mortality	5%	94	28.8%	66	38.9%	638	4.4	69

Abbreviations: LYs, life years; NNS, number needed to screen to prevent one cancer death; NNSurv, number needed to surveil; NNT, number needed to treat to prevent one cancer death.

^a^ Reduction of the PC incidence compared to no screening when PC risk is halved: 68, or doubled: 251.

^b^ Mortality reduction compared to no screening in case of halved PC risk: 55, or doubled PC risk: 207.

#### 3.3.1 PC risk

For Progressive‐only disease and annual screening, doubling the PC base case risk lowered the NNS from 431 to 225. If the base case PC risk was decreased by half, the NNS becomes 844. Similar changes were seen in the Indolent Included pathway (Figure 3; Appendix Table 2 and 3).

#### 3.3.2 Screen test performance

In the Progressive‐only pathway, lowering the test specificity from 90 to 85% led to an increase of the number of surveillance tests to prevent one cancer death (NNSurv), from 47 to 67, in case of annual screening. Increased specificity up to 100% decreased the NNSurv from 47 to 5. In the Indolent Included pathway, lowering of the test specificity from 90 to 85% led to an increase in NNSurv from 65 to 93, in case of annual screening. With 5‐yearly screening, the observed effect was smaller in both pathways.

For both pathways, an assumed 10% increase in test sensitivity for each disease stage lowered the PC incidence and mortality by <10%. The effect was smallest in case of annual screening.

#### 3.3.3 Treatment mortality

Varying treatment mortality from 3% to 5% resulted in an increase in PC‐related deaths of 4 to 8% for both disease pathways in both annual and 5‐yearly screening. It also modestly increased the NNS; for annual screening in the Progressive‐only pathway from 431 to 450, and in the Indolent Included pathway from 600 to 638.

## 4 DISCUSSION

Results from our model show that PC screening in high‐risk individuals is promising and may be worthwhile. Based on the results of our exploratory study, we conclude that the characteristics of the natural disease course (ie, probability of progression and average duration of preclinical disease stages) largely determines the efficacy of PC screening. Another important parameter we identified is the level of risk for developing PC. This is highly relevant, given the various genetic backgrounds of high‐risk individuals and the associated variable risk levels.

The sensitivity of the screening test has a much smaller effect on mortality reduction. The specificity of the test, however, is of particular importance for the number of surveillance tests (associated with burden) needed to prevent one PC death.

The influence of the test sensitivity on the NNS was negligible. This is partly caused by the fact that the probability that someone is referred for resection after a positive test was not varied (Appendix Table 1). Consequently, the change in resections (and LYG) was smaller than the change in screen positives. In case of a screening interval of 5 years and a 10% lower sensitivity, 8% more screening tests were needed to prevent one PC death compared to the base case sensitivity (Progressive‐only pathway). For the same comparison, the mortality rate was 4% lower. Furthermore, the low impact of the test sensitivity is influenced by the screening interval. Screening with a low test sensitivity can still be effective if the screen frequency is high. A screen test with a higher sensitivity could lead to a less intensive screening program with lower costs and a lower burden. Also, the specificity of PC screening tests could be much improved to decrease the number of false positive referrals and overtreatment. So, test development is still of much importance.

Microsimulation modeling is driven by available evidence and supplemented by assumptions. Ideally, these assumptions are based on high‐quality evidence. In case of screening for PC in high‐risk individuals, scientific data are still scarce. This paucity of high‐quality evidence can partly be explained by the relatively short period of time in which the effects of PC screening have been studied (the first report dates from 1999).^13^ Also, as opposed to other types of cancer, in which histologic samples from suspicious lesions can be easily obtained, this is not the case for pancreatic lesions. EUS‐guided tissue sampling, in particular of smaller lesions, is challenging and carries a risk of post‐procedural pancreatitis.

The design of this model and its input parameters are based on assumptions and data that are consistent with current knowledge in this field. Importantly, when new data becomes available, it can be incorporated in our model to improve its predictive value. Another strength of our study is that we have used a formal microsimulation model (in which the assumptions are well defined, can be reproduced and of which outputs can be compared to observations), and performed comprehensive sensitivity analyses, through which we have tested the robustness of our results. The MISCAN microsimulation model was developed by the Department of Public Health of the Erasmus MC (Rotterdam, the Netherlands) and has been extensively validated for the evaluation of multiple cancer screening programs (cancer of the breast, cervix, colorectal, lung, prostate and oesophagus).^2^, ^3^, ^4^, ^5^, ^6^, ^7^

In this exploratory analysis, the only included negative aspect of treatment was a 3% to 5% surgical mortality risk. Therefore, a potential limitation is that we omitted other negative aspects, such as morbidity and loss in quality of life, as a result of screening, surveillance or resection. These aspects need consideration, since pancreatic surgery is associated with significant morbidity (40%‐60%),^10^, ^11^ such as delayed gastric emptying, wound infections and pancreatic fistulae, in addition to diabetes mellitus and/or exocrine insufficiency as late complications. Integrating these harms in the model will likely result in a less favorable effect. Furthermore, the assumed 100% attendance rate for PC screening might have led to an overestimation on the population effect. However, literature shows that high risk individuals are likely to participate in PC screening.^14^ Also, we did not consider systematic false‐negative test results in our model. The sensitivity of a test is important, but repeated testing in a screening program generally enables missed lesions to be detected in a next screening‐round. However, some lesions may be systematically missed due to their size or (pre‐existing) parenchymal changes.

The NNS estimated for breast, cervical, colorectal and prostate cancer screening ranges from 1000 to 2000.^15^, ^16^ Our estimation that approximately 500 persons need to be screened to prevent one PC death is significantly lower. Although many of the factors that influence the NNS in our model are based on assumptions, none of the sensitivity analyses resulted in a NNS of >1500. Moreover, the screening instruments (ie, EUS, MRI) used to detect (preinvasive) PC are more invasive than those used in currently implemented screening programs. Also, they are more expensive (eg, approximately €700 for EUS,^17^ as compared to the <€60 spend by other screening programs^5^, ^7^, ^18^).

Few studies estimating the (cost‐)effectiveness of PC screening have been published.^17^, ^19^, ^20^, ^21^, ^22^ Pandharipande et al^21^ concluded that screening with MRI of individuals with even modestly increased risk may incur life expectancy gains. Rulyak et al^20^ found that EUS screening in high‐risk individuals increases patient life expectancy and is cost‐effective compared to no screening. Both studies involved a one‐time screening at the age of 50. Rubenstein et al^17^ concluded that for men with features of chronic pancreatitis, who are at high‐risk for developing PC, the most effective management strategy is no intervention. This may be due to the assumed high morbidity rates and loss in quality of life after total pancreatectomy.

To conclude, we showed that the natural disease course of PC and its precursor lesions is one of the determining factors of the success of pancreatic screening in high‐risk individuals. The risk for developing PC in the target population is another factor that plays a major role. Test sensitivity has a minor influence. Both base cases show that under plausible assumptions PC screening might be promising in a high‐risk population. The current study underlines the importance of continued research pertaining the development of PC and differential risks within specific target populations. This is of interest not only to improve test strategies based on imaging, but also on biomarkers in serum and secretin stimulated pancreatic juice.

## CONFLICT OF INTEREST

The authors disclosed no potential conflicts of interest.

## Supporting information


**Appendix** S1: Supporting information

## Data Availability

All data on which the model inputs of this analysis are based are available upon reasonable request to the corresponding author: b.koopmann@erasmusmc.nl.

## References

[1] Goggins M , Overbeek KA , Brand R , et al. Management of patients with increased risk for familial pancreatic cancer: updated recommendations from the International Cancer of the Pancreas Screening (CAPS) Consortium. Gut. 2020;69(1):7‐17. 31672839 10.1136/gutjnl-2019-319352 PMC7295005

[2] Habbema JDF , Van Oortmarssen GJ , Lubbe JTN , Van der Maas PJ . The miscan simulation program for the evaluation of screening for disease. Comput Meth Prog Bio. 1985;20(1):79‐93. 10.1016/0169-2607(85)90048-3 3849380

[3] Loeve F , Boer R , Zauber AG , et al. National polyp study data: evidence for regression of adenomas. Int J Cancer. 2004;111(4):633‐639. 15239144 10.1002/ijc.20277

[4] Lansdorp‐Vogelaar I , van Ballegooijen M , Boer R , Zauber A , Habbema JDF . A novel hypothesis on the sensitivity of the fecal occult blood test results of a joint analysis of 3 randomized controlled trials. Cancer. 2009;115(11):2410‐2419. 19288570 10.1002/cncr.24256 PMC2872543

[5] de Kok IMCM , van Rosmalen J , Dillner J , et al. Primary screening for human papillomavirus compared with cytology screening for cervical cancer in European settings: cost effectiveness analysis based on a Dutch microsimulation model. Brit Med J. 2012;e670:344. 10.1136/bmj.e670 PMC3293782 22391612

[6] Draisma G , Boer R , Otto SJ , et al. Lead times and overdetection due to prostate‐specific antigen screening: estimates from the European Randomized Study of Screening for Prostate Cancer. J Natl Cancer Inst. 2003;95(12):868‐878. 12813170 10.1093/jnci/95.12.868

[7] Saadatmand S , Tilanus‐Linthorst MM , Rutgers EJ , et al. Cost‐effectiveness of screening women with familial risk for breast cancer with magnetic resonance imaging. J Natl Cancer Inst. 2013;105(17):1314‐1321. 23940285 10.1093/jnci/djt203

[8] Kamarajah SK , Burns WR , Frankel TL , Cho CS , Nathan H . Validation of the American Joint Commission on Cancer (AJCC) 8th edition staging system for patients with pancreatic adenocarcinoma: a Surveillance, Epidemiology and End Results (SEER) analysis. Ann Surg Oncol. 2017;24(7):2023‐2030. 28213792 10.1245/s10434-017-5810-x

[9] Yachida S , Jones S , Bozic I , et al. Distant metastasis occurs late during the genetic evolution of pancreatic cancer. Nature. 2010;467(7319):1114‐1117. 20981102 10.1038/nature09515 PMC3148940

[10] Sohn TA , Yeo CJ , Cameron JL , et al. Resected adenocarcinoma of the pancreas ‐ 616 patients: results, outcomes, and prognostic indicators. J Gastrointest Surg. 2000;4(6):567‐579. 11307091 10.1016/s1091-255x(00)80105-5

[11] Winter JM , Cameron JL , Campbell KA , et al. 1423 pancreaticoduodenectomies for pancreatic cancer: a single‐institution experience. J Gastrointest Surg. 2006;10(9):1199‐1210. 17114007 10.1016/j.gassur.2006.08.018

[12] Bilimoria KY , Bentrem DJ, Ko CY, Ritchey J, Stewart AK, Winchester DP, et al. Validation of the 6th edition AJCC Pancreatic Cancer Staging System: report from the National Cancer Database. Cancer. 2007;110(4):738‐744. 10.1002/cncr.22852 17580363

[13] Brentnall TA , Bronner MP , Byrd DR , Haggitt RC , Kimmey MB . Early diagnosis and treatment of pancreatic dysplasia in patients with a family history of pancreatic cancer. Ann Intern Med. 1999;131(4):247‐255. 10454945 10.7326/0003-4819-131-4-199908170-00003

[14] Konings ICSG , Harinck F , Aalfs CM , et al. Repeated participation in pancreatic cancer surveillance by high‐risk individuals imposes low psychological burden. Psychooncology. 2016;25:971‐978. 26632416 10.1002/pon.4047

[15] Schroder FH . Stratifying risk—the U.S. Preventive Services Task Force and prostate‐cancer screening. N Engl J Med. 2011;365(21):1953‐1955. 22029756 10.1056/NEJMp1112140

[16] Hewitson P , Glasziou P , Watson E , Towler B , Irwig L . Cochrane systematic review of colorectal cancer screening using the fecal occult blood test (hemoccult): an update. Am J Gastroenterol. 2008;103(6):1541‐1549. 18479499 10.1111/j.1572-0241.2008.01875.x

[17] Rubenstein JH , Scheiman JM , Anderson MA . A clinical and economic evaluation of endoscopic ultrasound for patients at risk for familial pancreatic adenocarcinoma. Pancreatology. 2007;7(5–6):514‐525. 17912015 10.1159/000108969

[18] Heijnsdijk EA , Wever EM , Auvinen A , et al. Quality‐of‐life effects of prostate‐specific antigen screening. N Engl J Med. 2012;367(7):595‐605. 22894572 10.1056/NEJMoa1201637 PMC4982868

[19] Peters MLB , Eckel A , Mueller PP , et al. Progression to pancreatic ductal adenocarcinoma from pancreatic intraepithelial neoplasia: results of a simulation model. Pancreatology. 2018;18(8):928‐934. 30143405 10.1016/j.pan.2018.07.009 PMC9201992

[20] Rulyak SJ , Kimmey MB , Veenstra DL , Brentnall TA . Cost‐effectiveness of pancreatic cancer screening in familial pancreatic cancer kindreds. Gastrointest Endosc. 2003;57(1):23‐29. 12518126 10.1067/mge.2003.28

[21] Pandharipande PV , Heberle C , Dowling EC , et al. Targeted screening of individuals at high risk for pancreatic cancer: results of a simulation model. Radiology. 2015;275(1):177‐187. 25393849 10.1148/radiol.14141282 PMC4372492

[22] Pandharipande PV , Jeon A , Heberle CR , et al. Screening for pancreatic adenocarcinoma in BRCA2 mutation carriers: results of a disease simulation model. EBioMedicine. 2015;2(12):1980‐1986. 26844277 10.1016/j.ebiom.2015.11.005 PMC4703708

