# Supplementary material for: Identifying key factors for the effectiveness of pancreatic cancer screening: A model‐based analysis
Source: Int J Cancer. 2021 Mar 25;149(2):337–46. doi: 10.1002/ijc.33540 (PMC8251934; doi:10.1002/ijc.33540)

APPENDIX

Identifying Key Factors For The Effectiveness Of Pancreatic Cancer Screening: A Model-based Analysis

AUTHORS - Brechtje D.M. Koopmann, MD, Femme Harinck, MD PhD, Sonja Kroep, PhD, Ingrid C.A.W. Konings, MD PhD, Steffie K. Naber, PhD, Iris Lansdorp-Vogelaar, PhD, Paul Fockens, MD PhD, Jeanin E. van Hooft, MD PhD MBA, Djuna L. Cahen MD PhD, Marjolein van Ballegooijen, MD PhD, Marco J. Bruno, MD PhD, Inge M.C.M. de Kok, PhD

<u>Table of Contents</u>

Table 1. Assumptions for the sensitivity analyses on the sensitivity of the screening test and the follow-up strategy……………………………………………………………………..… Page 2

Table 2. Results of the sensitivity analyses for screening at ages 50 to 75, with a 1 and 5 year interval in the Progressive-only pathway……………………..……….………….Page 4

Table 3. Results of the sensitivity analyses for screening at ages 50 to 75, with a 1 and 5 year interval in the Indolent Included pathway………………………………………..….Page 5

Figure 1. Schematic overview of the natural history in the MISCAN-pancreas model………………………………………………………………………………………..Page 6

Table 1. Assumptions for the sensitivity analyses on the sensitivity of the screening test and the follow-up strategy (i.e. management). See Figure 2. Transition 1 (‘back to screening’) is the effect of (false-) negative test result.

	True stage	1. Back to screening	2. Surveillance, 6 months follow up	3. Resection
Base case	Normal	90%	10%	0%
	Preinvasive stage LGD	85%	12%	3%
	Preinvasive stage IGD	68%	17%	15%
	Preinvasive stage HGD	26%	44%	30%
	Preclinical I	10%	9%	81%
	Preclinical II	7%	7%	86%
	Preclinical III/IV	0%	0%	100%*
Sensitivity -10%	Normal	90%	10%	0%
	Preinvasive stage LGD	86%	11%	3%
	Preinvasive stage IGD	62%	24%	14%
	Preinvasive stage HGD	44%	33%	23%
	Preclinical I	19%	8%	73%
	Preclinical II	16%	6%	78%
	Preclinical III/IV	0%	0%	100%*
Sensitivity -5%	Normal	90%	10%	0%
	Preinvasive stage LGD	86%	11%	3%
	Preinvasive stage IGD	60%	26%	14%
	Preinvasive stage HGD	30%	41%	29%
	Preclinical cancer I	14%	9%	77%
	Preclinical cancer II	12%	6%	82%
	Preclinical cancer III/IV	0%	0%	100%*
Sensitivity +5%	Normal	90%	10%	0%
	Preinvasive stage LGD	84%	13%	3%
	Preinvasive stage IGD	66%	22%	12%
	Preinvasive stage HGD	33%	40%	27%
	Preclinical cancer I	5%	10%	86%
	Preclinical cancer II	2%	7%	91%
	Preclinical cancer III/IV	0%	0%	100%*
Sensitivity +10%	Normal	90%	10%	0%
	Preinvasive stage LGD	83%	14%	3%
	Preinvasive stage IGD	54%	29%	17%
	Preinvasive stage HGD	9%	54%	37%
	Preclinical cancer I	1%	10%	89%
	Preclinical cancer II	0%	7%	93%
	Preclinical cancer III/IV	0%	0%	100%*

All screen positives to resection	Normal	90%	10%	0%
	Preinvasive stage LGD	40%	0%	60%
	Preinvasive stage IGD	40%	0%	60%
	Preinvasive stage HGD	25%	0%	75%
	Preclinical cancer I	10%	0%	90%
	Preclinical cancer II	7%	0%	93%
	Preclinical cancer III/IV	0%	0%	100%*
≥ Preinvasive stage HGD to resection	Normal	90%	10%	0%
	Preinvasive stage LGD	40%	60%	0%
	Preinvasive stage IGD	40%	60%	0%
	Preinvasive stage HGD	25%	0%	75%
	Preclinical cancer I	10%	0%	90%
	Preclinical cancer II	7%	0%	93%
	Preclinical cancer III/IV	0%	0%	100%*
≥ Cancer to resection	Normal	90%	10%	0%
	Preinvasive stage LGD	40%	60%	0%
	Preinvasive stage IGD	40%	60%	0%
	Preinvasive stage HGD	25%	75%	0%
	Preclinical cancer I	10%	0%	90%
	Preclinical cancer II	7%	0%	93%
	Preclinical cancer III/IV	0%	0%	100%*

*In case of cancer stage III/IV, 99% of the patients only receives palliative care (no resection)

Table 2. Results of the sensitivity analyses for screening at ages 50 to 75, with a 1 and 5 year interval in the Progressive-only pathway. The interval cancer rate is presented as the number of cancer cases (per 100,000 LYs) in the first 5 years after a negative screening test and in the total period after a negative screening test (including at age 75). NNS = Number of screening tests need to perform to prevent one cancer death, NNT = Number needed to treat to prevent one cancer death, NNSurv = Number of surveillance test need to performed to prevent one cancer death.

Progressive-only pathway	Cancer cases prevented (per 10,000 persons)	Cancer deaths prevented (per 10,000 persons)	Incidence (per 100,000 LYs)	Mortality (per 100,000 LYs)	Interval cancers <5 years (per 100,000 LYs)	All Interval cancers (per 100,000 LYs)	NNS	NNT	NNSurv
NO SCREENING			132	108	n.a.	n.a.	n.a.	n.a.	n.a.
**Sensitivity analyses for screening annually from age 50 to 75**									
BASE CASE	454	433	67	46	25	40	431	2.9	47
Sensitivity +5%	461	437	66	45	23	38	427	2.8	46
Sensitivity +10%	470	442	64	44	21	36	422	2.8	46
Sensitivity -5%	445	427	68	46	27	43	437	2.9	47
Sensitivity -10%	436	421	69	47	30	45	443	2.9	48
Specificity 85%	459	435	66	45	25	40	429	2.8	67
Specificity 95%	451	431	67	46	24	39	433	2.8	26
Specificity 100%	447	429	68	46	24	39	435	2.9	5
Risk Halved	240	225	34	23	12	20	844	2.8	87
Risk Doubled	822	800	130	89	49	79	225	3.0	26
Increased preclinical stage	472	444	64	44	13	21	416	4.5	49
Decreased preclinical stage	371	371	77	53	52	76	505	2.3	52
All screen positives resection	588	494	47	37	13	26	381	2.9	39
≥Preinv stage HGD screen positives resection	538	480	55	39	20	42	392	2.2	45
≥Cancer screen positives resection	-133	168	151	84	53	77	1087	4.8	133
Treatment mortality 5%	455	414	67	48	25	40	450	3.0	49
**Sensitivity analyses for screening every 5 year, from age 50 to 75**									
BASE CASE	198	256	104	71	123	136	166	3.3	19
Sensitivity +5%	206	266	102	70	114	127	160	3.3	19
Sensitivity +10%	216	274	101	68	106	120	155	3.3	18
Sensitivity -5%	187	246	105	72	133	146	172	3.3	20
Sensitivity -10%	178	235	106	74	143	156	180	3.4	21
Specificity 85%	199	257	103	71	123	136	165	3.3	27
Specificity 95%	195	255	104	71	123	136	167	3.3	12
Specificity 100%	192	253	104	71	123	137	168	3.3	4
Risk Halved	105	134	53	36	62	69	323	3.2	35
Risk Doubled	345	470	200	137	244	269	87	3.4	12
Increased preclinical stage	189	304	105	64	77	85	139	4.8	19
Decreased preclinical stage	144	176	110	81	196	214	242	2.8	26
All screen positives resection	329	325	85	61	89	101	131	3.3	14
≥Preinv stage HGD screen positives resection	185	248	105	72	123	139	172	3.0	19
≥Cancer screen positives resection	-93	84	146	96	190	207	502	6.1	60
Treatment mortality 5%	198	247	103	72	123	136	172	3.4	20

Table 3. Results of the sensitivity analyses for screening at ages 50 to 75, with a 1 and 5 year interval in the Indolent Included pathway. The interval cancer rate is presented as the number of cancer cases (per 100,000 LYs) in the first 5 years after a negative screening test and in the total period after a negative screening test (including at age 75). NNS = Number of screening tests need to perform to prevent one cancer death, NNT = Number needed to treat to prevent one cancer death, NNSurv = Number of surveillance test need to performed to prevent one cancer death.

Indolent Included pathway	Cancer cases prevented (per 10,000 persons)	Cancer deaths prevented (per 10,000 persons)	Incidence (per 100,000 LYs)	Mortality (per 100,000 LYs)	Interval cancers <5 years (per 100,000 LYs)	All Interval cancers (per 100,000 LYs)	NNS	NNT	NNSurv
NO SCREENING			132	108	n.a.	n.a.	n.a.	n.a.	n.a.
**Sensitivity analyses for screening annually from age 50 to 75**									
BASE CASE	259	309	94	64	79	106	600	4.1	65
Sensitivity +5%	268	316	93	63	74	101	587	4.1	64
Sensitivity +10%	277	323	92	62	69	97	575	4.1	62
Sensitivity -5%	249	302	96	65	85	112	615	4.2	66
Sensitivity -10%	241	295	97	66	91	118	630	4.2	68
Specificity 85%	264	311	94	63	80	107	596	4.1	93
Specificity 95%	256	307	95	64	79	105	605	4.1	36
Specificity 100%	249	303	96	64	78	105	613	4.2	7
Risk Halved	137	161	48	33	41	55	1178	4.1	122
Risk Doubled	460	569	182	123	157	206	314	4.3	37
Increased preclinical stage	308	360	82	51	39	62	514	4.6	58
Decreased preclinical stage	165	226	117	84	153	178	817	4.7	87
All screen positives resection	419	399	71	51	51	77	471	3.7	48
≥Preinv stage HGD screen positives resection	374	381	78	53	67	97	492	2.8	58
≥Cancer screen positives resection	-201	132	161	89	106	133	1385	5.6	173
Treatment mortality 5%	260	291	94	66	79	106	638	4.4	69
**Sensitivity analyses for screening every 5 year, from age 50 to 75**									
BASE CASE	31	130	127	89	237	257	326	6.0	38
Sensitivity +5%	37	136	126	89	230	250	311	5.9	37
Sensitivity +10%	44	141	125	88	224	244	299	5.8	36
Sensitivity -5%	24	124	128	90	244	264	340	6.1	40
Sensitivity -10%	18	119	129	91	251	270	356	6.2	41
Specificity 85%	34	132	127	89	237	256	321	5.9	53
Specificity 95%	29	128	128	90	237	257	331	6.1	23
Specificity 100%	918	753	128	90	238	257	336	6.1	8
Risk Halved	16	67	65	46	120	130	639	6.0	69
Risk Doubled	53	242	242	171	467	501	167	6.0	22
Increased preclinical stage	49	196	119	75	151	170	215	5.8	27
Decreased preclinical stage	-13	71	142	106	329	346	590	8.2	67
All screen positives resection	137	189	112	81	194	214	224	5.4	24
≥Preinv stage HGD screen positives resection	44	134	125	89	235	255	314	4.8	36
≥Cancer screen positives resection	-171	48	156	101	281	299	870	8.4	107
Treatment mortality 5%	32	120	127	91	237	257	353	6.5	41

Figure 1. Schematic overview of the natural history in the MISCAN-pancreas model.

(A) Natural history of a progressive lesion

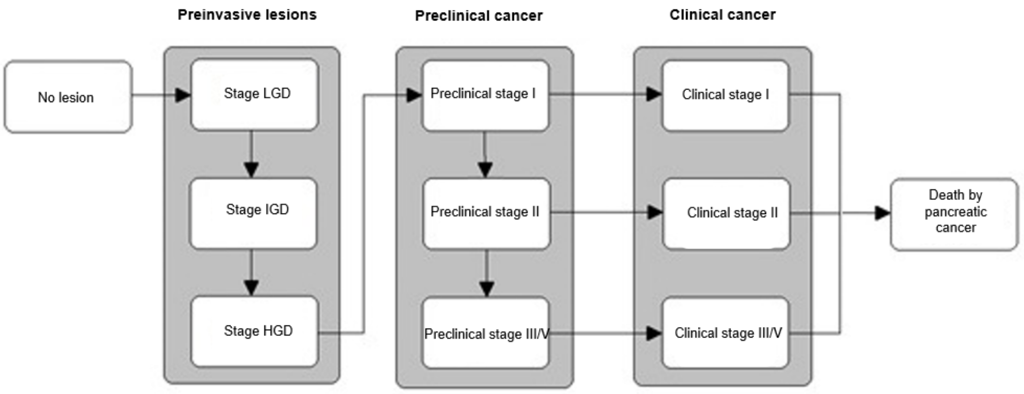

(B) Natural history of an indolent lesion

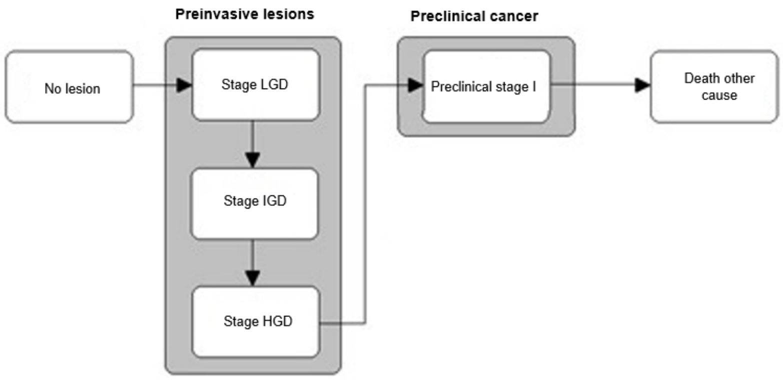

Supplement: Supplementary file 1 — Appendix S1: Supporting information [file IJC-149-337-s001.pdf]
